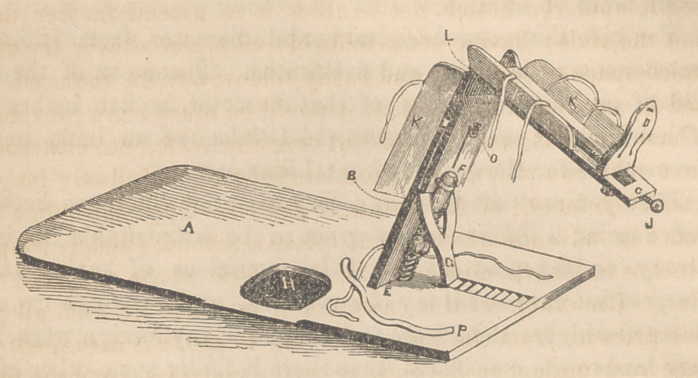# Apparatus for the Treatment of Fractures of the Inferior Extremities

**Published:** 1851-10

**Authors:** D. H. Agnew

**Affiliations:** 139 N. 11th St., Philadelphia; Lancaster Co., Pa.


*Apparatus for the treatment of fractures of the inferior extremities.* By D. H. AGNEW, M. D., of Lancaster Co., Pa.

In originating an apparatus for the treatment of fractures, there are several considerations which should claim attention, the most prominent of which, are:

The decubitus most comfortable for a protracted confinement; the position in which the limb should be placed in order to diminish or remove muscular contraction, one great cause of displacement; variety of adaptation, so that the same splint might be applicable to a number of casualties, or fulfil a diversity of indications, and simplicity of construction. Annexed is furnished the cut of a splint which the writer has had constructed, and which, upon trial, he thinks will be found to answer all the ends to be had in view in the management of fractures of the inferior extremities.

EXPLANATION.

A is a board 28 or 30 inches in width, and 3 feet 6 inches in length; B is a board 17 inches in length, 7 inches in width, tapering upward to 6¼ inches, and secured by a couple of stout hinges at E. O represents a second, notched 3 inches from its upper extremity, sufficiently deep to make the plane of the internal surface correspond with that of B, and extending down along the back of the latter 8 inches, with a narrow slit cut out, through which extends a rod, with a thumb-screw working on its extremity, in order to keep it accurately in contact with B. C is a board either cut out in its centre, or otherwise deeply excavated, secured to O by a hinge L 26 inches long and 6 inches wide, tapering forward to 4½ inches. D is a foot-board. F a female screw, secured to A and O. G, a forked support hinged to B, and maintaining it at any angle or inclination by working in the rachets seen in front. H an oval piece, cut out of A, beneath which is to be placed a bed pan. There should be another on the opposite side. The opening as represented in the cut, is a little too high up, and also too near the edge of the board A. J a small wood-screw fastened to the foot-board D. K K K, sides or wings secured to *b* and *c* by hinges. P a stout piece of webbing to surround the pelvis.

*Application.*—Place the apparatus upon a common bed stand with a sloat bottom, or if none such be at hand, upon any firm place as a settee, or even the floor, blocking up the body board A, to any inclination desired; throw over it either a mattress or several folds of coverlets, turning in the edge (if the latter be used) diagonally, so as to leave a spot over the slide H uncovered. A second may be folded so as to fill up the vacancy; (the object, it will be readily seen, is to have a portion moveable in order to accommodate, when necessary, a bed pan.) If the mattrass be used it would be better not to allow it to extend farther down than the slide; having some folds of quilts placed over the unoccupied space to level up, and easily removed when required. If used in any public institution, it would be much more convenient to have the mattrass blocked out over the slide, on either side. Next take two pillows, or a soft stuffed pad, sufficiently long to extend the length of the splints B and C. Upon the splint *c*, 2 inches in advance of the joint, place a second, (wedge shaped) 8 inches in length, and 1 or 2 inches thick at the end next the joint. The design of this compress or pad is to take off all possible pressure from the ham. Having things thus arranged, you place your patient on his back upon the mattrass, bend the thigh at an angle with the body, and place it against the splint B, then bringing the leg at a right angle with the thigh, place it upon the splint C. Next take a roller, and passing it round the foot and ankle in the form of the figure 8, until it attains a considerable thickness, stick securely to it on either side two good stout pieces of tape, and fasten the same to the foot-board *D*. By means of the transverse strap P you secure the pelvis to the mattrass, after which knot the other straps, taking care to interpose between each and the limb a soft compress to take off pressure. Having matters thus adjusted, should it be necessary to make traction upon the fragments of the os femoris with a view to prevent overlapping, commence turning the screw *F*, which by lengthening the splint any degree of force whatever, may be exerted. Or suppose you have a very oblique fracture of the tibia and fibula, in consequence of which, it is necessary to apply antagonistic forces to secure the proper length, all the alteration necessary is to place the wedge shaped compress beneath the thigh, a short distance above the popliteal space, the thick end uppermost, and by simply turning the screw *J* make any degree of extension requisite. Or imagine a fracture of both femur and bones of the leg, and in consequence of unequal degrees of resistance, requiring unequal degrees of force, in order to keep them in a proper position: how easily may this desideratum be accomplished without any interference whatever. Any lateral displacements are effectually prevented by closing up the sides of the splints K, K, K, which are attached by hinges as in the old fracture box, and interposing between the wings and the limb bags filled with bran.

The principle, it will be seen from the above explanation, is to make the body, by its weight, act as the counter-extending force, and the leg, as the point of extension in fractures of the femur; and in fractures of the tibia and fibula the thigh that of the former, and the foot of the latter force.

We think the following advantages may be claimed for this over other splints. A position of the body and limb attended with the greatest comfort for a prolonged confinement, and one calculated to obtain the greatest amount of muscular relaxation. The variety of postures or angles at which either the body or limbs may be placed, the effect of which change, however slight, being always a source of relief to a patient. Our being able to dispense with the perineal band, which so frequently produces troublesome excoriations, especially in cases of children and females; also avoiding chaffing the ankle, which occurs when it becomes necessary to apply a strong extending force. The ease with which evacuations from the bowels, may be received and removed without disconcerting the limb. Its applicability to a number of fractures occurring in the same limb, and the absence of all necessity for changing and readjustment.

P. S. Any medical gentlemen desirous of testing the apparatus may be furnished with one, by addressing Mr. Gildea, No. 61 Dock St., Philadelphia.

139 N. 11th St., Philadelphia.

## Figures and Tables

**Figure f1:**